# Ginkgolide B increases hydrogen sulfide and protects against endothelial dysfunction in diabetic rats

**DOI:** 10.3325/cmj.2015.56.4

**Published:** 2015-02

**Authors:** Guo-Guang Wang, Qing-Ying Chen, Wei Li, Xiao-Hua Lu, Xue Zhao

**Affiliations:** 1 Department of Pathophysiology, Wannan Medical College, Wuhu, People’s Republic of China; 2 General Hospital of Jinan Military Command, Jinan, People’s Republic of China; * Contributed equally to the study.

## Abstract

**Aim:**

To evaluate the effect of ginkgolide B treatment on vascular endothelial function in diabetic rats.

**Methods:**

The study included four groups with 15 male Sprague-Dawley rats: control group; control group treated with ginkgolide B; diabetic group; and diabetic treated with ginkgolide B. The activity of superoxide dismutase (SOD), malondialdehyde content, and nicotinamide adenine dinucleotide phosphate (NADPH) oxidase subunits, and glutathione peroxidase 1 (GPX1) protein expression were determined in aortic tissues. Vasoconstriction to phenylephrine (PHE) and vasorelaxation to acetylcholine (Ach) and sodium nitroprusside (SNP) were assessed in aortic rings. Nitric oxide (NO) and hydrogen sulfide (H_2_S) were measured, as well as cystathionine γ lyase (CSE) and cystathionine β synthetase (CBS) protein expression, and endothelial nitric oxide synthase (eNOS) activity.

**Results:**

Diabetes significantly impaired PHE-induced vasoconstriction and Ach-induced vasorelaxation (*P* < 0.001), reduced NO bioavailability and H_2_S production (*P* < 0.001), SOD activity, and GPX1 protein expression (*P* < 0.001), and increased malondialdehyde content and NADPH oxidase subunits, and CSE and CBS protein expression (*P* < 0.001). Ginkgolide B treatment improved PHE vasoconstriction and Ach vasorelaxation (*P* < 0.001), restored SOD (*P* = 0.005) and eNOS (*P* < 0.001) activities, H_2_S production (*P* = 0.044) and decreased malondialdehyde content (*P* = 0.014). Vasorelaxation to SNP was not signiﬁcantly different in control and diabetic rats with or without ginkgolide B treatment. Besides, ginkgolide B increased GPX1 protein expression and reduced NADPH oxidase subunits, CBS and CSE protein expression.

**Conclusion:**

Ginkgolide B alleviates endothelial dysfunction by reducing oxidative stress and elevating NO bioavailability and H_2_S production in diabetic rats.

Diabetes mellitus is an endocrine disease caused by decreased insulin secretion or action, leading to impaired glucose and lipid metabolism (1). The most dangerous complication of diabetes mellitus is cardiovascular disease, which is the primary factor leading to high mortality and morbidity in diabetic patients (2). A critical role in diabetic cardiovascular complications is played by endothelial dysfunction. Mechanisms responsible for endothelial dysfunction are still poorly understood, but hyperglycemia-induced oxidative stress is hypothesized to be one of them (3). Increased blood glucose increases reactive oxygen species (ROS) production via glucose auto-oxidation (4) and variation in activity of oxidoreductases, such as NADPH oxidase (5). ROS can impair vascular function by damaging endothelial cells, thus playing an important role in diabetes and its cardiovascular complications (6). NADPH oxidase is important because it generates ROS (7,8). Glutathione peroxidase 1 (GPX1) is one of the pivotal antioxidant enzymes in vascular endothelium, which protects against the presence of coronary artery disease (9). Its overexpression reduces ROS formation and enhances phosphorylation of endothelial nitric oxide synthase (eNOS), which improves endothelial function (10).

Hydrogen sulfide (H_2_S) was previously considered only as a toxic gas, but recent studies have suggested that it plays a variety of important physiological and physiopathological roles (11,12). H_2_S is generated from L-cysteine by several enzymes including cystathionine γ lyase (CSE) and cystathionine β synthetase (CBS). Some studies have shown that H_2_S takes part in modulation of cardiovascular system (13,14). It has also been shown that H_2_S biosynthesis is impaired in diabetes, and that it may be effective to administer different H_2_S donors to diabetic animals (15,16).

The risk of endothelial dysfunction is increased by sustained progression of hyperglycemia and hyperlipidemia. Endothelial dysfunction is characterized by alterations of endothelium-dependent vascular response to vasoconstrictors and vasodilators in diabetic animals (17,18). Therefore, cardiovascular complications including endothelial dysfunction in patients with diabetes have been treated by decreasing blood glucose and lipid content, and reducing activation of angiotensin. However, these treatments have not prevented the development of complications (19), emphasizing the need for novel approaches.

Ginkgolide B, a plant-derived terpenoid, is one of natural bioactive components from the extract of ginkgo biloba leaves. Many studies have demonstrated that ginkgolide B can inhibit platelet-activating factor (PAF)-induced platelet activation via binding with PAF receptor (20,21). Therefore, ginkgolide B is widely used as a natural antagonist of PAF and inhibitor of PAF-induced inflammatory reaction (22). It has been shown that ginkgolide B regulates many physiologic functions, including the antioxidant function, improving the cognitive functions of central nervous system (23,24), repressing atherosclerosis (25), and abating liver cirrhosis (26). In this study, we investigated the effects of ginkgolide B on endothelial function and mediators such as hydrogen sulfide (H_2_S), biomarkers of oxidative stress, and oxidoreductase in the aorta of rats with streptozotocin-induced diabetes.

## Methods

### Experimental animals

Sixty 8-10 weeks old male Sprague-Dawley rats (180-220 g) were purchased from the central animal laboratory of Wannan Medical College. All experimental procedures were approved by the Academic Experimental Animal Care and Use Committee of Wannan Medical College. Animals received a standard pellet diet and water *ad libitum*, and were raised for 2 weeks in a standard animal laboratory at 22 ± 2°C temperature and with a 12-hour daylight cycle for acclimatization before the experiment.

After 12 hours of fasting, thirty rats were intraperitoneally injected STZ (65 mg/kg) dissolved in 0.1 mol/L natrium citricum buffer (pH 4.5) for induction of diabetes mellitus. Thirty control rats were injected with the same volume buffer. All rats received a standard chow and water. After 72 hours of STZ treatment, blood was drawn from the tail vein to measure the levels of the fasting blood glucose for confirmation of diabetes mellitus. Rats with the fasting glucose values >15 mmol/L were regarded as diabetic and used for further experiments.

### Experimental protocol and treatment

After diabetes was confirmed, control and diabetic rats were randomly divided into four groups as follows: a) control group (CG, n = 15): rats received standard food and water; b) control treated group (CT, n = 15): rats were treated with 5 mg/kg/d ginkgolide B and received standard food and water; c) diabetic group (DG, n = 15): rats received standard food and water; d) diabetic treated group (DT, n = 15): rats were treated with 5 mg/kg/d ginkgolide B and received standard food and water. Ginkgolide B was given orally by gavage for 8 weeks.

### Biochemical analyses of blood samples

At the end of the experiment, body weight was measured, and blood specimens were collected into prechilled test tubes. To assess the change of blood glucose and serum lipid profiles, blood specimens were centrifugated at 1300 × g for separation of serum. Concentrations of serum lipid profiles were determined by the enzymatic colorimetric methods (commercial kits from Nanjing Jiancheng Bioengineering Institute, Nanjing, China).

### *Measurement of oxidative stress, eNOS activity, and nitric oxide* (*NO) production*

After eight weeks of ginkgolide B treatment, rats were anesthetized with sodium pentobarbital and euthanized, and blood samples and aortic tissues were immediately collected. For oxidative stress analysis, aortic tissue was homogenized and centrifugated at 13 000 × g for 10 minutes at 4°C. The supernatants were used to analyze redox enzyme activity (superoxide dismutase) and peroxidation production of lipid (malonaldehyde). eNOS activity and NO production in the supernatant were determined by enzymatic colorimetric methods (commercial kits from Nanjing Jiancheng Bioengineering Institute).

### Determination of H_2_S

H_2_S production in the aorta tissue was determined as previously described (27). Aorta tissues were collected and homogenized in an ice-cold lysis buffer (potassium phosphate buffer 100 mmol/L pH7.4, Na3VO4 10 mmol/L). Homogenates (250 μL) were mixed with 20 μL pyridoxal 5′-phosphate, 20 μL L-cysteine, and 30 μL normal saline in sealed Eppendorff tubes and incubated at 37şC. CCl3COOH (10%, 250 μL), zinc acetate (1%, 250 μL), and 0.5 mL borate buffer (pH 10.01) were placed one by one in the tubes after 40 minutes. The mixture was incubated with N,N-dimethylphenylendiamine sulfate (20 mM, 133 μL) in 7.2 mol/L HCl and FeCl_3_ (30 mmol/L, 133 μL) in 1.2 mol/L HCl at 37°C for 30 minutes. The H_2_S production was determined by measuring the absorbance of final solution at 670 nm. Plasma determination of H_2_S was performed without addition of L-cysteine according to above-mentioned method.

Vascular reactivity to vasoconstrictor and vasodilator were measured in aortic rings, which are commonly used as a model of vasomotoricity (28). Briefly, after animals were sacriﬁced, the thoracic aortas were immediately harvested, and blood and surrounding connective tissue was cleared away in the plate filled with Krebs-Henseleit buffer (KH buffer, pH 7.4) with sodium chloride (118 mmol/L), potassium chloride (4.7 mmol/L), monopotassium phosphate (1.2 mmol/L), magnesium sulfate (1.2 mmol/L), sodium bicarbonate (25 mmol/L), glucose (5.5 mM), and calcium chloride (2.5 mmol/L). The aortas were carefully cut into 3-4 mm aortic rings. The endothelial layer was removed in certain experiments so that the effects of the endothelium on vascular responses can be observed. The arterial rings were fixed in the tissue chamber with KH buffer with access to 95% O_2_ and 5% CO_2_ gas mixtures at 37°C, and were connected to tension sensor and stainless wire in the bottom of the chamber.

After 60 minutes equilibration, concentration-dependent vasoconstriction to phenylephrine (PHE, α-adrenoceptor agonist) was observed. To estimate the effect of ginkgolide B on the endothelium-dependent relaxation, vessels were preconstricted with 10^−6^ mol/L PHE, and concentration-dependent vascular responses to acetylcholine (Ach) and sodium nitroprusside (SNP, nitric oxide donor) were examined. The integrity of the endothelium was verified by determining the endothelium-dependent vasodilation to Ach after contraction to PHE (10^−6^ mol/L). To determine the effects of eNOS and cyclooxygenase, vascular rings were pre-incubated in the KH buffer with or without N^ω^-nitro-l-arginine methyl-ester (L-NAME, non-specific NOS inhibitor, 100 μmol/L) or methylene blue (MB, cGMP inhibitor, 10 μmol/L) for 20 minutes before the addition of PHE. The changes of contract tension to PHE were recorded. Vascular relaxation to Ach and SNP was expressed as percentage from submaximal constriction to PHE.

### Western blotting

To analyze expression of redox enzyme, aortic tissues were harvested and homogenized in a prechilled buffer with Tris-HCl (20 mmol/L), Na_3_VO_4_ (1 mmol/L), Na_4_P_2_O_7_ (2.5 mmol/L), phenylmethylsulfonyl fluoride (2 mmol/L), EDTA (1 mmol/L), EGDA (1 mmol/L), and protease inhibitor mixture (10 μg/mL). Homogenates were centrifugated. Proteins in the supernatants were electrophoretically separated on 8% stacking gel and following 12% separation gel of sodium dodecyl sulfate-polyacrylamide gel electrophoresis transferred onto nitrocellulose membrane. Membranes were incubated with blocking buffer containing Tris-HCl (20 mmol/L), NaCl (500 mmol/L), Tween-20 (0.1%), non-fat milk (5%) to block non-specific sites and incubated with rabbit polyclonal CBS, CSE, GPX-1, NOX-2, NOX-4, β-actin antibody dissolved in the blocking buffer (1:500) overnight at 4ºC. Membranes were incubated with horseradish peroxidase-conjugated anti-rabbit IgG antibody after 3 washing steps (20 mmol/L Tris, 500 mmol/L NaCl, 0.1% Tween-20) for 1 hour. After having been rinsed with wash buffer for three times, the reaction was visualized by DAB.

### Statistical analysis

The values are presented as means ± standard error of the mean. Differences between the groups were analyzed with one way analysis of variance (ANOVA) using least significance difference and *t* test. The analysis was performed with SPSS, 16.0 (SPSS Inc., Chicago, IL, USA). A *P* value of less than 0.05 was considered statistically significant.

## Results

### Animal characteristics

Diabetic and diabetic treatment groups showed significantly higher blood glucose levels than control groups (*P* < 0.001), which did not change after ginkgolide B treatment (Table 1). Diabetic group had lower body weight than control group, and ginkgolide B significantly alleviated the decline in diabetic group (*P* < 0.001) (Table 1). Diabetic group had higher levels of total cholesterol, triglycerides, and low density lipoprotein (LDL) and lower levels of high-density lipoprotein (HDL) than control group (*P* < 0.001) (Table 1). Ginkgolide B treatment significantly reduced cholesterol (*P* < 0.001), triglycerides (*P* = 0.029), and LDL (*P* = 0.011) concentrations, but elevated HDL (*P* = 0.003) concentration in diabetic rats (Table 1).

**Table 1:** Effects of ginkgolide B (5 mg/kg) on biochemical profile (n = 10 per group)*

	Control group	Control treatment group	Diabetic group	Diabetic treatment group
Body weight (g)	331 ± 10.4	327 ± 11.2	179 ± 11.7^†^	228 ± 15.9^‡^
Blood glucose (mmol/L)	4.63 ± 0.58	4.76 ± 0.61	26.31 ± 3.77^†^	24.53 ± 3.14
Total cholesterol (mmol/L)	2.19 ± 0.47	1.97 ± 0.36	6.20 ± 0.82^†^	4.16 ± 0.72^‡^
Triglycerides (mmol/L)	1.51 ± 0.37	1.43 ± 0.35	3.05 ± 0.88^†^	2.18 ± 0.48^‡^
Low density lipoprotein (mmol/L)	2.32 ± 0.45	2.11 ± 0.42	5.28 ± 1.22^†^	3.56 ± 0.62^‡^
High-density lipoprotein (mmol/L)	1.14 ± 0.20	1.19 ± 0.12	0.47 ± 0.12^†^	0.68 ± 0.17^‡^

*Results are expressed as mean ± standard error of the mean.

†*P* < 0.001 compared with control group.

‡*P* < 0.05 compared with diabetic group, in particular *P* < 0.001 compared with diabetic group for body weight and total cholesterol, *P* = 0.029 for triglycerides, *P* = 0.011 for low density lipoprotein, *P* = 0.003 for high-density lipoprotein.

### Antioxidant effects of ginkgolide B

**Figure 1:**
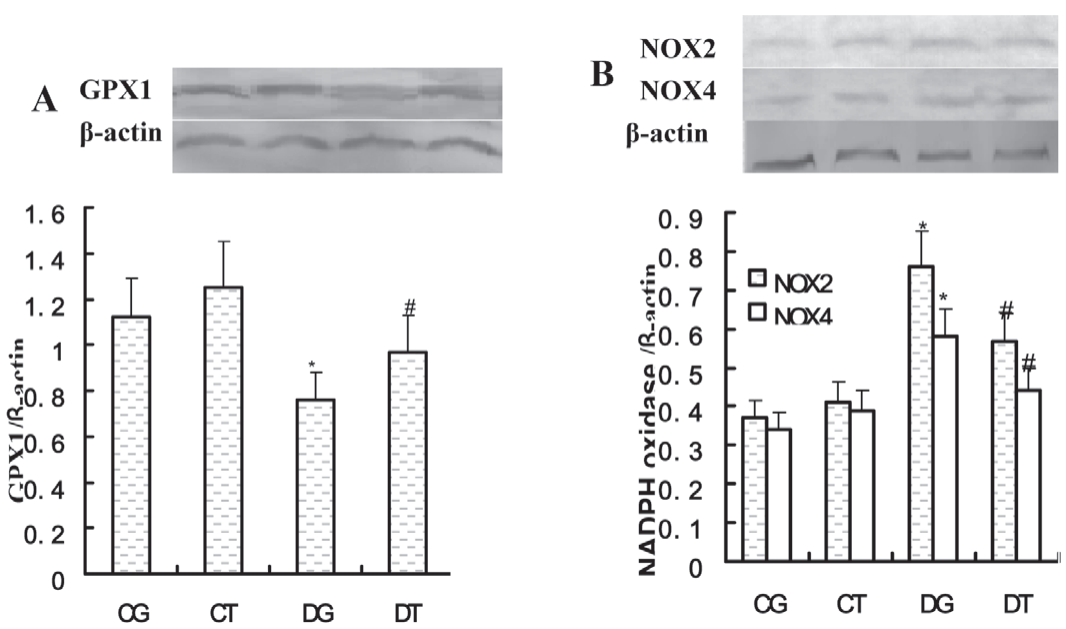
Glutathione peroxidase 1 (GPX 1) and nicotinamide adenine dinucleotide phosphate (NADPH) oxidase protein expression in aortas of control and diabetic rats treated or not treated with ginkgolide B (5 mg/kg). (**A**) Representative Western blots (upper panel) and quantitative analysis (lower panel) for GPX 1; (**B**) Representative Western blots (upper panel) and quantitative analysis (lower panel) for NOX2 and NOX4. CG – control group; CT – control treated group; DG – diabetic group; DT – diabetic treated group. Results are expressed as mean ± standard error of the mean. The number of animals per group was 8. *P < 0.05 compared with control group; #*P* < 0.05 compared with diabetic group.

The activity of SOD was decreased (137.09 ± 10.46 vs 193.69 ± 11.80 U/mg, *P* < 0.001) and malondialdehyde content was increased (2.02 ± 0.33 vs 1.23 ± 0.13 nmol/mg, *P* < 0.001) in diabetic compared with control rats. Ginkgolide B significantly decreased malondialdehyde content (1.64 ± 0.24 vs 2.02 ± 0.33 nmol/mg, *P* = 0.014) and elevated SOD activity (153.10 ± 8.68 vs 137.09 ± 10.46 U/mg, *P* = 0.005) in diabetic rats (Table 2). Oxidases Nox2 and Nox4 protein expression was significantly elevated in diabetic groups compared with control rats (Figure 1B), while antioxidase GPX 1 level was reduced (Figure 1A). Ginkgolide B decreased the expression of Nox2 and Nox4 proteins and increased expression of GPX 1 protein in diabetic rats (Figure 1A, 1B).

**Table 2:** Superoxide dismutase (SOD) activity and malondialdehyde (MDA) content in aortas of control and diabetic rats treated with or without ginkgolide B (5 mg/kg). Data are expressed as mean ± standard deviation (n = 8 per group)

	Control group	Control treatment group	Diabetic group	Diabetic treatment group
Activity of SOD(U/mg protein)	193.69 ± 11.80	190.41 ± 11.17	137.08 ± 10.46*	153.10 ± 8.68^†^
Content of MDA(nmol/ mg protein)	1.23 ± 0.13	1.17 ± 0.14	2.02 ± 0.33*	1.62 ± 0.24^†^

*P < 0.0001, compared with control group.

^†^*P* < 0.05 compared with diabetic group.

### Effect of ginkgolide B on NO production and NOS activity

NO production was decreased (2.62 ± 1.02 vs 8.43 ± 0.93 μmol/g, *P* < 0.001) and eNOS activity was reduced (232.27 ± 24.44 vs 565.34 ± 56.97 U/g, *P* < 0.001) in diabetic rats compared with control rats. Ginkgolide B significantly improved NO production (6.34 ± 1.36 vs 2.62 ± 1.02 μmol/g, *P* < 0.001) and reduced eNOS activity (495.99 ± 59.06 vs 232.27 ± 24.44 U/g, *P* < 0.001) in diabetic rats (Table 3).

**Table 3:** Effects of ginkgolide B on nitric oxide (NO) production and activity of endothelial nitric oxide synthase (NOS) in control and diabetic rats treated with or without ginkgolide B (5 mg/kg). Data are expressed as mean ± standard deviation (n = 8 per group)

	Control group	Control treatment group	Diabetic group	Diabetic treatment group
NO production (μmol/g protein)	8.43 ± 0.93	8.26 ± 1.26	2.62 ± 1.02*	6.34 ± 1.36^†^
NOS activity (U/g protein)	565.3 ± 57.0^†^	552.5 ± 77.3^†^	232.3 ± 24.4^†^	495.99 ± 59.1^†^

*P < 0.0001, compared with control group.

†*P* < 0.001 compared with diabetic group.

### Effect of ginkgolide B on vascular reactivity

**Figure 2:**
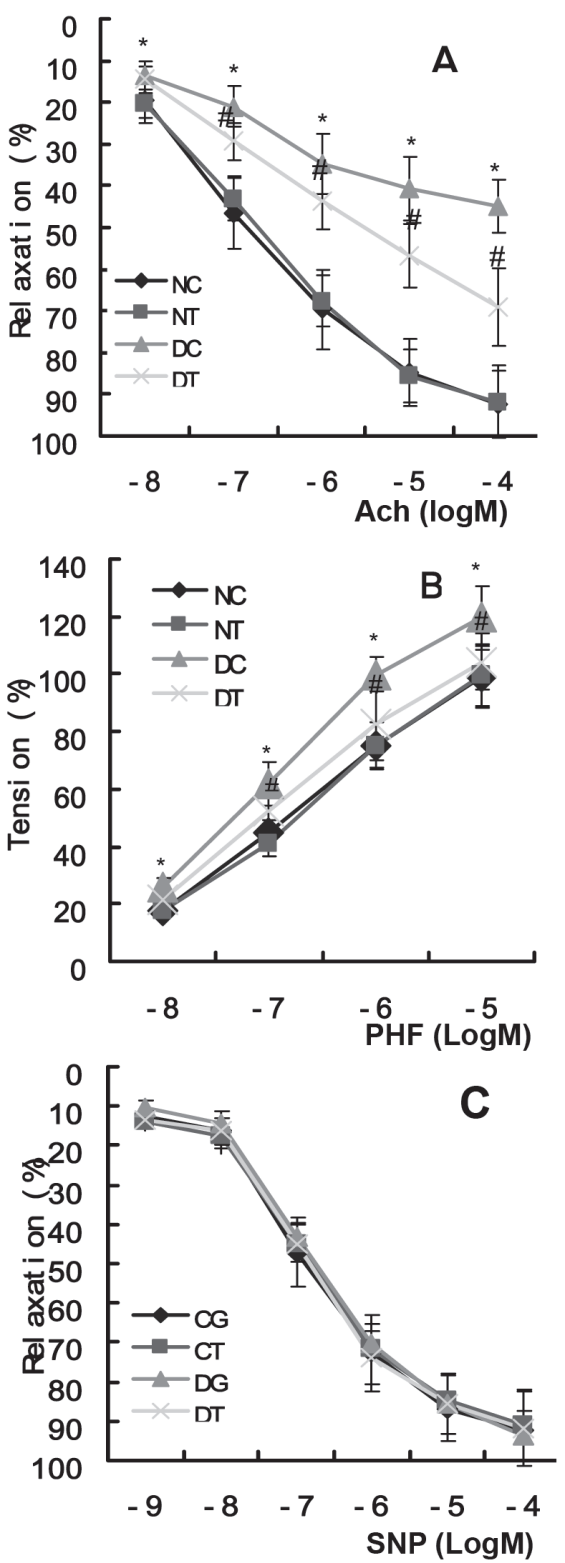
Effects of ginkgolide B on vascular reactivity to acetylcholine (**A**), phenylephrine (**B**), and sodium nitroprusside (SNP) (**C**). CG – control group; CT – control treated group; DG – diabetic group; DT – diabetic treated group. *P < 0.05 compared with control group; #*P* < 0.05 compared with diabetic group. Data are expressed as mean ± standard deviation. n = 8 rats for each group.

Diabetic rats had reduced vasorelaxation to Ach compared with control rats (the maximum response [Rmax]: 44.8 ± 6.4 vs 92.2 ± 7.9%, *P* < 0.001) (Figure 2A), but there were no signiﬁcant differences in vasorelaxation to SNP between all groups (Rmax: 93.7 ± 7.5 vs 90.8 ± 6.2%, *P* = 0.691) (Figure 2C). Vasoconstriction to PHE was higher in diabetic than in control rats (Rmax: 120.0 ± 9.7 vs 98.8 ± 5.4%, *P* < 0.001) (Figure 2B). The impaired vasorelaxation to Ach was improved (Rmax: 69.1 ± 9.4 vs 98.8 ± 5.4%, *P* < 0.001) and the increased vasoconstriction to PHE was reduced in diabetic rats compared with diabetic treatment rats (Rmax: 104.2 ± 10.6 vs 120.0 ± 9.7%, *P* = 0.007). Vasodilatations to Ach and vasoconstriction to PHE were not changed in control treatment rats.

### Effects of ginkgolide B on endothelial, cGMP, and NO modulation of PHE-induced contraction

In all groups except diabetic group, vascular rings incubated with L-NAME or MB showed an increase in maximum response to PHE compared with not-incubated vascular rings. L-NAME or MB did not change the maximum response to PHE in diabetic controls (Table 4). Absence of endothelial cells increased the maximum responses to PHE compared with the presence of endothelial cells, and maximum vasoconstriction to PHE was not different among all the groups in the absence of endothelial cells (Table 5).

**Table 4:** Effects of ginkgolide B on the maximum response to PHE incubated with or without L-NAME or MB (5 mg/kg) (n = 8 per group)*†

		PHE (mol/L) induced contraction response (%)			
		10^−8^	10^−7^	10^−6^	10^−5^
Control group	No incubation	17.89 ± 2.25	44.73 ± 4.52	75.13 ± 10.12	98.77 ± 5.35
	L-NAME	26.92 ± 3.91^‡^	72.06 ± 7.18^§^	107.89 ± 12.79^§^	126.80 ± 10.87^§^
	MB	55.70 ± 5.61^§^	108.13 ± 14.62^§^	123.29 ± 12.63^§^	146.91 ± 10.25^§^
Control treatment group	No incubation	17.44 ± 2.01	40.83 ± 4.12	78.71 ± 6.81	99.60 ± 11.02
	L-NAME	29.92 ± 4.63^ǁ^	70.80 ± 6.87^ǁ^	104.88 ± 9.27^ǁ^	127.67 ± 11.48^ǁ^
	MB	52.28 ± 6.49^ǁ^	102.92 ± 13.85^ǁ^	116.15 ± 12.05^ǁ^	146.16 ± 12.27^ǁ^
Diabetic group	No incubation	25.69 ± 3.39^‡^	61.89 ± 7.56^§^	99.77 ± 12.65^§^	120.04 ± 9.69^§^
	L-NAME	33.10 ± 6.49^††^	68.04 ± 7.13	105.64 ± 12.15	126.82 ± 11.01
	MB	51.54 ± 8.37^††^	94.52 ± 12.35^††^	112.23 ± 11.58**	134.89 ± 10.84
Diabetic treatment group	No incubation	21.23 ± 3.30**	52.59 ± 7.74^††^	82.35 ± 5.95^††^	104.21 ± 8.58^††^
	L-NAME	35.51 ± 3.13^¶^	71.95 ± 4.14^¶^	123.21 ± 8.30^¶^	126.66 ± 11.31^¶^
	MB	58.08 ± 6.63^¶^	102.30 ± 7.41^¶^	118.63 ± 8.76^¶^	144.56 ± 11.01^¶^

*L-NAME – L-*N*^G^-nitro-l-arginine methyl ester; MB – methylene blue; PHE – phenylephrine. No incubation – vascular rings were pre-incubated in the KH buffer without L-NAME and MB for 20 min before the addition of PHE; L-NAME – vascular rings were pre-incubated in the KH buffer with L-NAME for 20 min before the addition of PHE; MB – vascular rings were pre-incubated in the KH buffer with MB for 20 min before the addition of PHE.

†Results are expressed as mean ± standard error of the mean.

‡*P* < 0.05 or §*P* < 0.01 compared with control group (no incubation).

ǁ*P* < 0.01 compared with control treatment (no incubation).

¶*P* < 0.01 compared with diabetic treatment (no incubation).

**P < 0.05 or ††*P* < 0.01 compared with diabetic group (no incubation).

**Table 5:** Effects of ginkgolide B and endothelial removal on the maximum response to phenylephrine (PHE) (5 mg/kg) (n = 8 per group)*

	Control group	Control treatment group	Diabetic group	Diabetic treatment group
Endothelium intact (g)	0.81 ± 0.20	0.86 ± 0.32	1.45 ± 0.30^†^	1.09 ± 0.23^‡^
Endothelium removed (g)	2.59 ± 0.44^§^	2.53 ± 0.48^§^	2.40 ± 0.34^§^	2.43 ± 0.37^§^

*Results are expressed as mean ± standard error of the mean.

†*P* < 0.01 compared with control group.

‡*P* < 0.05 compared with diabetic group.

§*P* < 0.01 compared with endothelium intact ring.

### Effect of ginkgolide B on H_2_S

**Figure 3:**
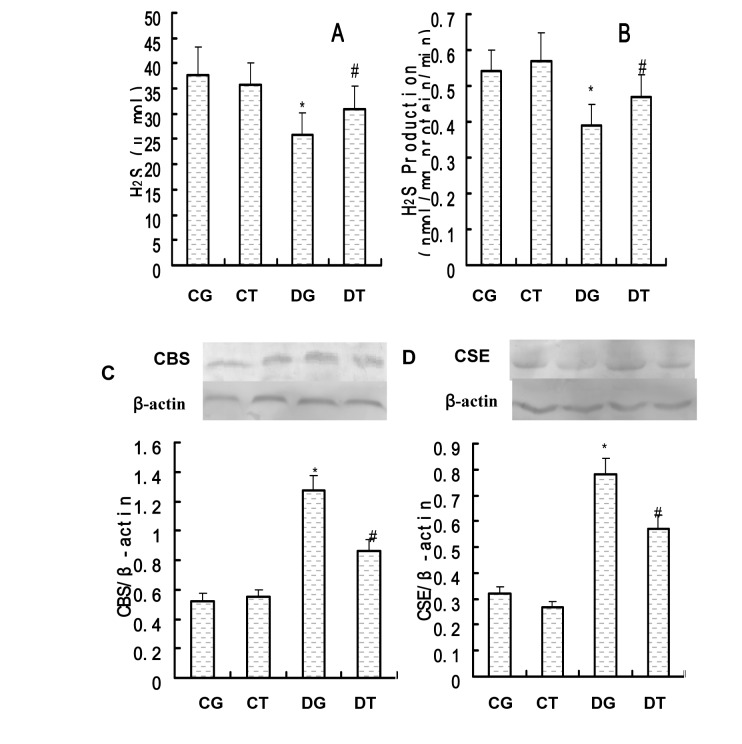
Effects of ginkgolide B on hydrogen sulfide (H_2_S) content in plasma, H_2_S production and cystathionine β synthetase (CBS) and cystathionine γ lyase (CSE) protein expression in the artery. (**A**) H_2_S plasma levels; (**B**) H_2_S production (**C**) Representative Western blots (upper panel) and quantitative analysis (lower panel) for CBS; (**D**) Representative Western blots (upper panel) and quantitative analysis (lower panel) for CSE. CG – control group; CT – control treated group; DG – diabetic group; DT – diabetic treated group. Results are expressed as mean ± standard error of the mean. The number of animals per group was 8. *P < 0.05 compared with control group; #*P* < 0.05 compared with diabetic group.

Diabetic rats exhibited a progressive reduction in H_2_S plasma levels (Figure 3A) and diabetes significantly impaired the ability to convert L-cysteine into H_2_S (Figure 3B). Ginkgolide B significantly increased H_2_S plasma levels in diabetic rats (Figure 3A) and enhanced the ability to convert L-cysteine into H_2_S (Figure 3B). Western blot analysis was performed to evaluate the effect of ginkgolide B on expression of CBS and CSE. The expression of CBS and CSE was significantly up-regulated in diabetic rats compared with control rats (Figure 3C, 3D). However, the expression of CBS and CSE was significantly down-regulated in diabetic treatment rats compared with diabetic rats (Figure 3C, 3D).

## Discussion

In this study, we provide evidence that ginkgolide B attenuated the progressive endothelial and vascular dysfunction in diabetic rats by increasing antioxidant and improving vascular regulation and prevented the development of vascular dysfunction by increasing the expression of CBS and CSE.

Ginkgolides are terpenoids from the extract of ginkgo biloba leaves, which are widely used as natural antagonist of PAF to reduce platelet activation (29). Ginkgolide B exhibited the strongest inhibition of platelet activation (20,21). Hyperglycemia impairs vasorelaxation and vasoconstriction by damaging vascular endothelial cells (30,31). Increased levels of serum lipids in diabetes increase the risk of complications (32). A previous study showed that ginkgolide B ameliorated plasma cholesterol and/or HDL levels (29), while this study showed that it decreased the levels of cholesterol, triglycerides, and LDL, and elevated HDL levels in diabetic rats. HDL has been shown to exhibit cardioprotective effect (33) and sphingosine-1-phosphate, a constituent of HDL, improved endothelial function and caused NO-dependent vasorelaxation (34). Our results showed that diabetic rats had improved lipoperoxidation, as confirmed by increased malondialdehyde level and decrease in activity of antioxidant enzyme as SOD. Furthermore, we observed reduced antioxidase GPX 1 level and elevated oxidase Nox2 and Nox4 levels.

Ginkgolide B exhibits an antioxidant effect via scavenging peroxy radicals (28,29,35-37), as well as reduces reactive oxygen species and malondialdehyde levels, supporting its antioxidant role (38,39). We also observed that it down-regulated GPX1 and increased Nox2 and Nox4 protein expressions, as well as SOD activity in diabetic rats.

Recent studies have shown that ginkgolide B protects endothelial cells and inhibits atherosclerosis plaque (25,40). Similarly, our study showed that it improved both vasorelaxation to Ach and vasoconstriction to PHE in diabetic rats, which may be related to increase in eNOS activity and NO production. NO is a critical factor for regulation of vascular responses, and endothelial dysfunction may be related to the decrease in NO production in STZ-induced diabetic rats (41). We also observed that the endothelium-independent vasorelaxation to SNP was not significantly different between diabetic and control groups, and that ginkgolide B treatment did not change vasorelaxation. Furthermore, we assessed the impact of L-NAME (eNOS inhibitor) or MB (cGMP inhibitor) pretreatment on vasoconstriction to PHE. The maximum response to PHE was increased in control, treatment control, and diabetic treatment group, but it was not changed in the diabetic group. These results suggested that ginkgolide B improved endothelial function possibly by reducing impairment of endothelium and enhancing eNOS activity. Therefore, the mechanisms underlying the ability of ginkgolide B to prevent endothelial dysfunction could involve an enhanced NO bioactivity, leading to improved endothelium-dependant vascular responses.

A recent study suggested that the H_2_S pathway was involved in vascular dysfunction in diabetic mice (42). Our results showed that H_2_S level in diabetic rats was reduced, but that the expression of CBS and CSE protein was up-regulated, which is consistent with previous results (15). Furthermore, treatment with ginkgolide B increased H_2_S level and down-regulated the expression of CBS and CSE protein. H_2_S is known as a gasotransmitter with protective effects in various tissue injuries (42-44). It is easily oxidized by reaction with oxygen and free radicals (45,46). A recent study showed that H_2_S released from Na_2_S increased endothelial NO production through Akt activation and subsequently increased phosphorylation of eNOS at Ser1177 (47). Therefore, the increase in CBS and CSE protein expression can be a compensatory response resulting from oxidative stress induced by hyperglycemia, and oxidative stress-reduced H_2_S production. Ginkgolide B elevated H_2_S level by improving oxidative stress, which increased NO production.

In summary, this study demonstrated that treatment with ginkgolide B attenuated the progressive endothelial and vascular dysfunction in diabetic rats. The protective effect of ginkgolide B on endothelial function may result from its inhibition of oxidative stress and modulation of NO and H_2_S production in the aorta of diabetic rats.
